# Flavopereirine—An Alkaloid Derived from *Geissospermum vellosii*—Presents Leishmanicidal Activity In Vitro

**DOI:** 10.3390/molecules24040785

**Published:** 2019-02-21

**Authors:** João Victor da Silva e Silva, Helliton Patrick Cordovil Brigido, Kelly Cristina Oliveira de Albuquerque, Josiwander Miranda Carvalho, Jordano Ferreira Reis, Lara Vinhal Faria, Márlia Regina Coelho-Ferreira, Fernando Tobias Silveira, Agnaldo da Silva Carneiro, Sandro Percário, Andrey Moacir do Rosário Marinho, Maria Fâni Dolabela

**Affiliations:** 1 Post-Graduate Program in Pharmaceutical Sciences, Federal University of Pará, Belém, PA 66075-110, Brazil; jvssilva89@gmail.com (J.V.d.S.e.S.); kellyoalbuquerque@hotmail.com (K.C.O.d.A.); 2 Laboratory of Immunomodulation and Protozoology, Oswaldo Cruz Institute, FIOCRUZ, Rio de Janeiro, RJ 21040-900, Brazil; 3 Post-Graduate Program in Pharmaceutical Innovation, Federal University of Pará, Belém, PA 66075-110, Brazil; helitom2009@hotmail.com; 4 College of Chemistry, Federal University of Pará, Belém, PA 66075-110, Brazil; mcwander@hotmail.com (J.M.C.); andrey@ufpa.br (A.M.d.R.M.); 5 Faculty of Pharmacy, Federal University of Pará, Belém, PA 66075-110, Brazil; jordanoreis@outlook.com (J.F.R.); laravf24@gmail.com (L.V.F.); agsicar@yahoo.com.br (A.d.S.C.); 6 Emílio Goeldi Paraense Museum, Coordination of Botany, Ministry of Science, Technology, Innovation and Communications, Belém, PA 66077-530, Brazil; mcoelho@museu-goeldi.br; 7 Evandro Chagas Institute, National Health Foundation, BR-316 Highway km 7, Ananindeua, PA 67030-000, Brazil; fernandotobias@iec.pa.gov.br; 8 US Centers for Disease Control and Prevention (CDC), Atlanta, GA 30329, USA; percario@ufpa.br; 9 Oxidative Stress Research Lab, Institute of Biological Sciences (ICB), Federal University of Pará, Belém, PA 66075-110, Brazil

**Keywords:** leishmania, *Geissospermum vellosii*, apocynaceae, oligopeptidase B, flavopereirine, medicinal chemistry, in silico modeling

## Abstract

Chemotherapy is limited in the treatment of leishmaniasis due to the toxic effects of drugs, low efficacy of alternative treatments, and resistance of the parasite. This work assesses the in vitro activity of flavopereirine on promastigote cultures of *Leishmania amazonensis*. In addition, an in silico evaluation of the physicochemical characteristics of this alkaloid is performed. The extract and fractions were characterized by thin-layer chromatography and HPLC-DAD, yielding an alkaloid identified by NMR. The antileishmanial activity and cytotoxicity were assayed by cell viability test (MTT). The theoretical molecular properties were calculated on the Molinspiration website. The fractionation made it possible to isolate a beta-carboline alkaloid (flavopereirine) in the alkaloid fraction. Moreover, it led to obtaining a fraction with greater antileishmanial activity, since flavopereirine is very active. Regarding the exposure time, a greater inhibitory effect of flavopereirine was observed at 24 h and 72 h (IC_50_ of 0.23 and 0.15 μg/mL, respectively). The extract, fractions, and flavopereirine presented low toxicity, with high selectivity for the alkaloid. Furthermore, flavopereirine showed no violation of Lipinski’s rule of five, showing even better results than the known inhibitor of oligopeptidase B, antipain, with three violations. Flavopereirine also interacted with residue Tyr-499 of oligopeptidase B during the molecular dynamics simulations, giving a few insights of a possible favorable mechanism of interaction and a possible inhibitory pathway. Flavopereirine proved to be a promising molecule for its antileishmanial activity.

## 1. Introduction

Amazonian species belonging to the family Apocynaceae are characterized by the presence of alkaloids, many of which are used in traditional medicine in the treatment of hard-to-heal wounds [1]. Within this family, two genera have deserved special attention for possessing alkaloids that have been shown as promising antimalarial molecules: *Aspidosperma* and *Geissospermum*. However, there is a knowledge gap regarding the antileishmanial potential of these genera, with reports only describing the antileishmanial properties of *Aspidosperma ramiflorum* Muell. Arg. and *Geissospermum reticulatum* A. Gentry. The alkaloid extract of *A. ramiflorum* was active against promastigotes of *L.* (L.) *amazonensis* and *L.* (V.) *braziliensis* [2]. The extract was fractionated, isolating the alkaloids ramiflorines A and B. Both ramiflorines A and B showed activity against *Leishmania* (L.) *amazonensis* (LD_50_ 16.3 ± 1.6 μg/mL and 4.9 ± 0.9 μg/mL, respectively) [3].

*G. reticulatum* presented in its composition the indole alkaloids *O*-demethylaspidospermine, which was active against *L. infantum* (IC_50_ 7.7 μg/mL), with a smaller effect on *T. cruzi* (IC_50_ 41.7 μg/mL); and *N*-Deacetyl-*N*-butanoylgeissospermidine, which was only active against *L. infantum* (IC_50_ 52.2 μg/mL). When comparing the toxicity of these molecules against the reference drugs, amphotericin B and nifurtimox (CC_50_ 10.3 and 13.9 μg/mL, respectively), the cytotoxicity of *O*-demethylaspidospermine was lower than both of them (CC_50_ 16.7 μg/mL) when assayed using mammalian Chinese hamster ovaries (CHO). Therefore, *O*-demethylaspidospermine presented a better selectivity for Leishmania (Selectivity Index, SI = 2.17) [4].

**Figure 1:**
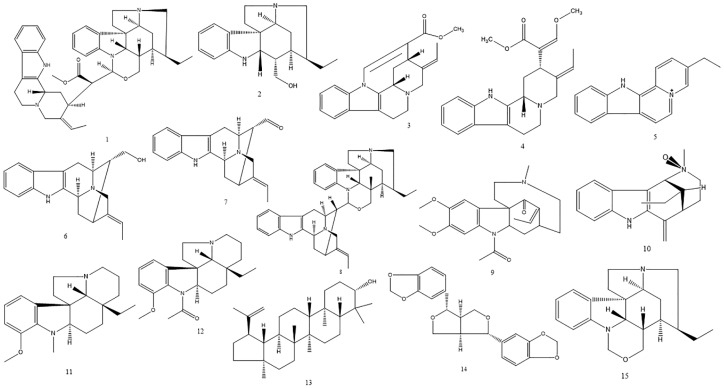
Main compounds isolated from *G. vellosii*. (1.1) Geissospermine [7]; (1.2) geissoschizoline; (1.3) apogeissoschizine; (1.4) geissoschizine [8]; (1.5) flavopereirine [9]; (1.6) vellosiminol; (1.7) vellosimine; (1.8) geissolosimine [10]; (1.9) geissovelline [11]; (1.10) pausperadine [12]; (1.11) 12-methoxy-1-methyl-aspidospermidine [13]; (1.12) aspidospermine; (1.13) lupeol; (1.14) sesamine [14]; (1.15) geissoschizone [5].

Phytochemical studies of the species *Geissospermum vellosii* Allemão (Apocynaceae) led to the isolation of several compounds, shown in Figure 1. The alkaloids isolated from *G. vellosii* were tested in clones of *Plasmodium falciparum* chloroquine-sensitive strain, obtaining potential antiplasmodial results for the indole alkaloids geissolosimine (IC_50_ 0.66 μg/mL), geissospermine (IC_50_ 0.65 μg/mL), geissoschizoline (IC_50_ 0.89 μg/mL), geissoschizone (IC_50_ 1.78 μg/mL), and vellosiminol (IC_50_ 1.04 μg/mL) [5]. Another study analyzed the activity against two strains of *P. falciparum* (chloroquine-resistant K1 and chloroquine-sensitive T9-96) with the indole alkaloids geissoschizoline and geissoschizoline N4-oxide presenting low selectivity (SI = 1; IC_50_ and CC_50_ > 40 μM). In addition, 1,2-dehydrogeissoschizoline showed a higher activity in the resistant clone (K1—CI_50_ 27.26 μM; T9-96—CI_50_ 35.37 μM), and the β-carboline alkaloid flavopereirine was more active in *P. falciparum* (K1—IC_50_ 11.53 μM; T9-96—IC_50_ 1.83 μM), with high selectivity for the sensitive parasite (SI = 5.85 for T9-96) [6]. Thus, among these alkaloids, flavopereirine was the most active tested compound. The antiplasmodial activity of *G. vellosii* was related to this alkaloid. However, no evaluation of the leishmanicidal activity for this alkaloid was found in the literature, and this evaluation was necessary.

The physicochemical properties of certain functional groups are fundamental in the pharmacodynamic phase of the mechanisms of action of many drugs. This is because during the molecular recognition stage, the sum of the interacting forces of the pharmacophoric groups of the ligand with the complementary sites of the receptor is an essential factor for the pharmacological effect of the medicine. The pharmacokinetic phase of absorption, distribution, metabolization, and excretion is also a direct result of the bioavailability and half-life of a drug, being directly affected by the variation of physicochemical properties. Therefore, studies that associate in silico results with in vitro experiments will certainly contribute to help elucidate the main characteristics of a potential drug [15]. Moreover, studies have shown that oligopeptidase B (OpB)—a cytosolic protein belonging to the prolyl oligopeptidase family of serine proteases (Clan SC, family S9) [16,17], common in trypanosomatids [18]—regulates enolase levels on the cell surface of parasites of the genus Leishmania, which contributes to the virulence of several infectious agents. The connection between OpB and the parasite suggests that it is a key point in the host infection [19], being a promising target for the development of new antileishmanial drugs. Other studies have shown that alkaloids can significantly inhibit the activity of oligopeptidases [20,21,22]. However, the inhibitory activity of various alkaloids has not yet been evaluated. That being said, this work evaluates in vitro studies of flavopereirine on promastigote cultures of Leishmania amazonensis as well as the in silico properties of flavopereirine.

## 2. Results

### 2.1. In Vitro Results

#### 2.1.1. *G. vellosii* Prospection and Phytochemical Profile Show the Presence of an Alkaloid

The ethanol extract obtained from barks of *G. vellosii* had a yield of 2.0% (Table 1). The extract was subjected to fractionation by extraction under reflux, resulting in four fractions. Of these, the methanol fraction showed the highest yield (85.2%; Table 1), indicating that the extract is rich in polar substances. Another method used for extract fractionation was the acid–base partition, yielding two fractions: neutral fraction (42.8%) and alkaloid fraction (27.5%; Table 1). This low yield of the alkaloid fraction suggests that the concentration of alkaloids in the extract is reduced.

**Figure 2:**
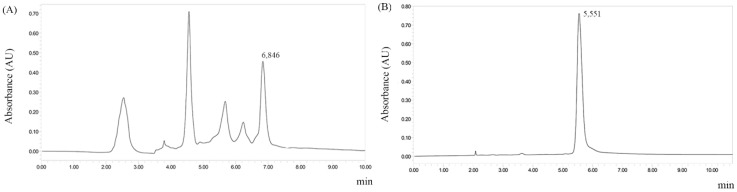
Chromatographic HPLC profiles of the subfraction F6AF and flavopereirine isolation of ethanolic extract from stem barks of *G. vellosii*. Condition: SunFire™ Prep. C18 OBD™ 5 µm 19 × 150 mm column, flow rate = 10.0 mL/min, temperature 18 °C. Mobile phase—t = 0 min: 80% water and 20% acetonitrile; t = 10 min: 80% water and 20% acetonitrile. (**A**) Chromatogram to subfraction F6AF; (**B**) flavopereirine-isolated *G. vellosii.*

Thin-layer chromatography studies suggest that the extract and its fractions should have alkaloids (Table 1). The alkaloid fraction (AF) was subjected to chromatographic fractionation on a Sephadex LH-20 column, yielding subfraction F6AF. HPLC-DAD studies of subfraction F6AF suggested the presence of alkaloids, which led to the fractionation of that subfraction by preparative HPLC, giving the alkaloid flavopereirine (Figure 2).

#### 2.1.2. The Alkaloid Derived from Flavopereirine Presents High Antipromastigote Activity

The antipromastigote activity of the extract, fractions, and alkaloid were evaluated at different times (24, 48, and 72 h). Most of the active samples showed better inhibitory effects at 24 h, and this effect was reduced at 48 h. The acid–base fractionation contributed to obtaining a very active fraction, the alkaloid fraction (AF). Further fractionation of this fraction yielded flavopereirine, which is probably the alkaloid responsible for such activity (Table 2).

The extract of *G. vellosii* underwent re-extraction under reflux. The hexane and ethyl acetate fractions were not promising as antileishmanial. Nevertheless, the methanol fraction was shown to be active, especially at 24 h. Fraction FrDcmalso presented better activity at 24 h. However, the antipromastigote effect appears to be reduced with increased exposure time (Table 2).

Subfraction F6AF showed up to be more active than the alkaloid fraction itself (t = 24 h). Notwithstanding, at 72 h, no significant difference was observed between them (*p* > 0.05). Flavopereirine displayed pronounced antileishmanial activity at all times (Table 2).

#### 2.1.3. Cytotoxicity and Selectivity Index of Flavopereirine Improved with Exposure Time in Comparison to Amphotericin B

Similar to the evaluation of antileishmanial activity, *G. vellosii* cytotoxicity was evaluated against modified THP-1 cells at different treatment times. A reduction of cytotoxicity with increased exposure time and no significant toxicity at 48 and 72 h of exposure (CC50 > 400 µg/mL) was observed. The extract, subfraction F6AF, flavopereirine, and amphotericin B proved to be very selective (SI > 10). When comparing the selectivity of flavopereirine over amphotericin B, it was observed that flavopereirine was more selective than amphotericin B, both at 24 h and 72 h (Table 3).

### 2.2. In Silico Results

#### 2.2.1. Flavopereirine Presented Better Theoretical Properties than Antipain

To understand this effect of flavopereirine, in silico studies were performed. The compiled results from the theoretical properties of the Molinspiration website [23] are shown in Table 4. Flavopereirine showed a rather theoretical lipophilic LogP (0.90; Table 4), while antipain presented a hydrophilic profile (−3.01; Table 4). In addition, flavopereirine presented no violation of Lipinski’s rule of five, which differs from antipain, with 3 violations (Table 4).

#### 2.2.2. Flavopereirine Appears to Inhibit OpB by Directly Binding to Tyr-499 in Silico

**Figure 3:**
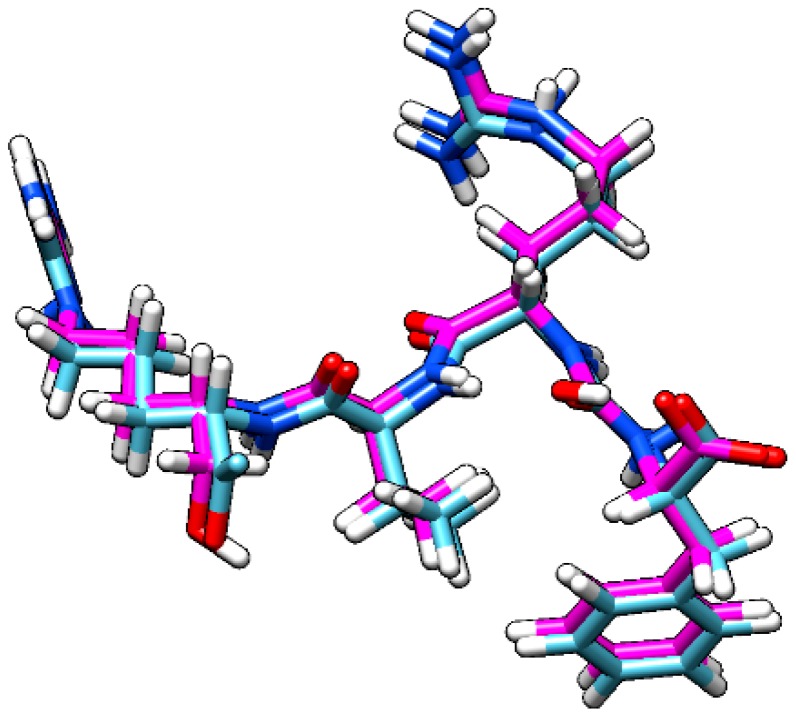
Docked antipain superposed with antipain from crystallography. In magenta, docked antipain; in blue, crystallographic antipain.

Figure 2 shows that our docking method was enough to ideally represent how antipain acts by inhibiting OpB, showing a RMSD of 0.3601 Å. We analyzed the best-docked pose of antipain superposed with crystallographic antipain (Figure 3). In this sense, the chosen methodology showed to be adequate for the subsequent computational simulations of flavopereirine.

**Figure 4:**
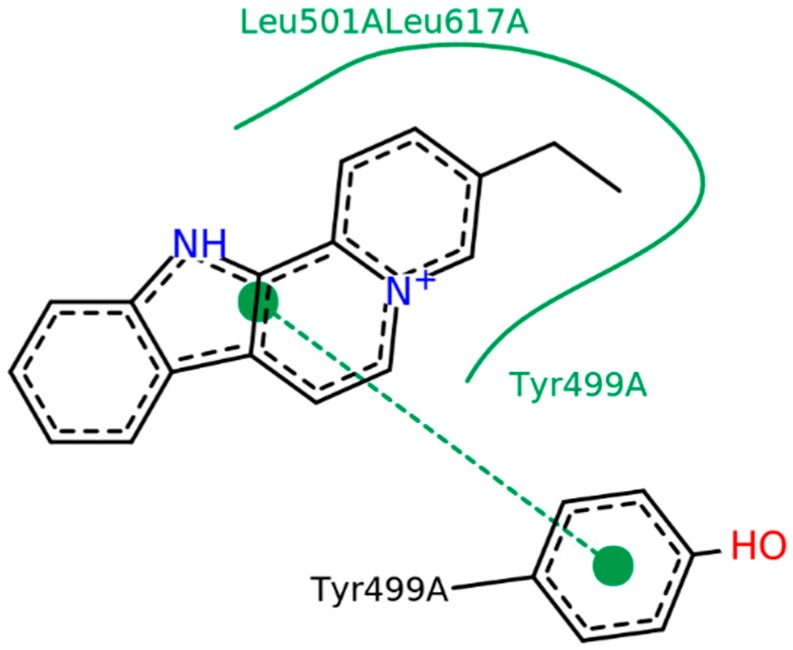
Intermolecular interactions between flavopereirine and OpB. In green outline, Van der Waals interactions; in dotted green line, π-stacking interactions. Image generated using PoseView (Version 1.1, BioSolveIT GmbH, St. Augustin, Germany) [25].

The bidimensional representation of our molecule docked in OpB is presented in Figure 3, showing the intermolecular interactions between both flavopereirine and OpB residues. It is possible to note that the hydrophobic interactions are the only ones present in the form of Van der Waals interactions (green outline) and π-stacking interactions (green dotted line) (Figure 4).

#### 2.2.3. Flavopereirine Shows High Affinity to OpB in the Molecular Dynamics Simulation

**Figure 5:**
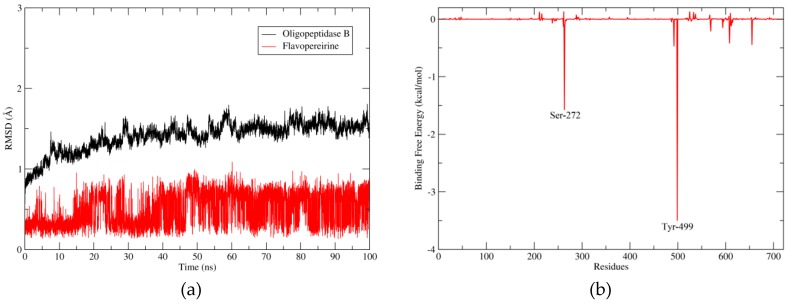
Molecular dynamics results. (**a**) RMSD x Time graphic of the molecular dynamics, with the black line representing the fluctuation of OpB and the red line the fluctuation of flavopereirine. (**b**) The main contributions to the binding free energy of each residue from OpB, mainly Ser-272 and Tyr-499.

The results of molecular dynamics are represented in Figure 5. Moreover, in Figure 5a, it is shown how the complex OpB-flavopereirine fluctuated through the molecular dynamics. Our results demonstrated that both OpB and flavopereirine fluctuated very little and stood in a very similar conformation to the structure at the beginning of the dynamics, having a RMSD lower than 2 Å. The free binding energy calculated by the MMPBSA method was −15.2747 kcal/mol. Figure 5b shows the individual contribution of each residue to the free binding energy.

A strong and attractive interaction between flavopereirine and residue Tyr-499, which is responsible for the abstract recognition of OpB, was computationally verified; this might be impeding the binding site and provoking the inactivation of this important enzyme of the parasite.

## 3. Discussion

The leishmanicidal activities of the alkaloid, extract, and fractions were evaluated after different exposure times. Flavopereirine and fractions containing the alkaloids (AF, F6AFGV, FDcm, and FMeOH) were very promising against promastigote forms of L. amazonensis (IC50 < 10 µg/mL), showing a rapid inhibitory effect on the parasite, as assessed after the first hours of exposure (Table 3). Extracts and fractions containing alkaloids were active against promastigote forms of *L. amazonensis*; however, flavopereirine was the most active. This fact was also observed in the literature evaluating its antiplasmodial in vitro activity against two strains of *P. falciparum* (multidrug-resistant clone K1 and chloroquine-sensitive T9-96; K1-IC50 11.53 μM and T9-96-IC50 1.83 μM) [6]. A very positive point observed in this study was that bioguided fractionation made it possible to gain more information about secondary metabolites, which may contribute to the leishmanicidal activity as well as to the improvement of selectivity (Table 3). This suggests that flavopereirine is the pharmacological marker of the activity observed for that species. Furthermore, it is worth noting that this is the first report on the leishmanicidal effects of flavopereirine. This beta-carbolic alkaloid has been shown to be more selective than amphotericin B, a drug that presents a complexity of factors (e.g., toxicity) that make treatment compliance difficult. Therefore, the search for therapeutic alternatives with less toxicity for leishmaniasis is very important.

Oligopeptidase B (OpB) is a cytosolic protein belonging to the prolyl oligopeptidase family of serine proteases (Clan SC, family S9) [16,17]. It is a protein common in trypanosomatids [18], being involved in the cleavage of peptides in the carboxyl region of basic residues, with preference for arginine or lysine residues [26,27]. With the in vitro results in hand, it is very important to clarify the possible inhibitory mechanisms of action of flavopereirine using in silico approaches. To start, we chose *L. major* OpB as a target because of the known correlation between alkaloids and oligopeptidase inhibition [21,28] and because of the known similarities of prolyl oligopeptidase and OpB [29]. Lipinski’s rule of five was initially applied to express the theoretical properties of flavopereirine and antipain, a potent inhibitor of OpB [30].

The compiled results from the theoretical properties demonstrated that flavopereirine did not present any violation of Lipinski’s rule of five (Table 4), showing drug-like characteristics with respect to bioavailability. The results also show that flavopereirine is more lipophilic than antipain, implying a better capacity of trespassing membranes and acting intracellularly, which is essential for inhibiting OpB, a cytosolic enzyme [31]. It is also observed, through Total Polar Surface Area (TPSA) values, that flavopereirine (19.89) has a greater transport capacity in the biological environment when compared to antipain (282.51) [32]. Still, according to the theoretical studies performed, from the tested molecules, flavopereirine presented itself as the most drug-like molecule by having no violations to Lipinski’s rule of five [33]. Lipinski has established that three or more violations of the rule could negatively impact the pharmacokinetics of any drug-aspiring molecule [24,33]. The incapability of antipain to cross membranes and its inhibitory effects on other peptidases and proteinases might be the reason for its non-use as a leishmanicidal agent.

The molecular docking results showed great interaction between flavopereirine and residue Tyr-499, an important residue for the abstract recognition of OpB [30]. The RMSD results from the molecular dynamics simulations showed a very stable binding to OpB, presented in Figure 5a with a graph lower than 1 Å throughout the entire simulation. Even though the ligand does not interact with any of the known residues of the canonical catalytic triad (Ser-577, His-697, Asp-662), this interaction with Tyr-499 might be strong enough to stabilize the complex [30]. Such interaction was emphasized according to the free binding energy results calculated using the Molecular Mechanics Poisson−Boltzmann/Surface Area (MM-PBSA) method. MM-PBSA is a simple but rather accurate end-state method of computing binding energy [34,35,36,37,38]. It showed a low energy, suggestive of a good interaction, which may be enough to prevent the OpB substrate to reach the catalytic site, and inhibiting the action of the enzyme.

## 4. Materials and Methods

### 4.1. Theoretical Molecular Properties

To calculate theoretical molecular properties, such as LogP, total polar surface area, number of hydrogen bond donors and acceptors, molecular weight, number of atoms, and number of rotatable bonds of flavopereirine, antipain, and amphotericin B, the Molinspiration website was used [23].

### 4.2. Molecular Docking

The tridimensional structure of oligopeptidase B (OpB) from Leishmania major was obtained from the Protein Data Bank (PDB), under the code 2XE4 [30]. Flavopereirine’s tridimensional structure was downloaded from PubChem (PubChem ID: 65171) [39]. To perform the molecular docking, Molegro Virtual Docker (MVD) software version 4.3.0 (CLC Bio, Aarhus, Denmark) was used [40]. The binding cavity from the known inhibitor of OpB, antipain, was used as a reference to proceed with the simulation. The MolDock score function was selected with a grid resolution of 0.20 Å and an interaction radius of 12 Å. The search algorithm used was “MolDock SE”. After performing 100 runs, there were 10 poses generated based on the interaction energy.

### 4.3. Molecular Dynamics Simulation

The best pose from the molecular docking was chosen as the starting conformation for the molecular dynamics studies. The electrostatic potentials (ESP) of flavopereirine were calculated at the Hartree–Fock level with the 6-31G* basis set, using Gaussian 09 (Gaussian, Inc., Wallingford, CT, USA) [41]. Flavopereirine was parametrized by assigning the restrained electrostatic potential (RESP) using the Antechamber and ParmEd.py modules of theAmberTools13 package [42]. All missing hydrogens were added using the TLEaP module [42]. Using the same module, TIP3P water molecules were added in an octahedral box of 8 Å surrounding the complex, and as many counterions were added as necessary [43].

Energy minimization and molecular dynamics simulations were performed using Amber 12 packages and PMEMD software (Version 12.0, University of California, San Francisco, CA, USA) [42]. All minimizations included a total of 5000 cycles. For the first 3000 cycles, we used the steepest-descent algorithm [44], and for the last 2000 cycles, we used the conjugate gradient algorithm [45]. During all minimization steps, a cut-off of 8 Å was defined for intermolecular interactions. After the minimization, the system was gradually heated from 0 to 300 K in six steps at 25 ps each, summing 150 ps and restricting the binding site. With the system heated properly, molecular dynamics was performed using general amber force field (gaff) [46] for flavopereirine, and ff12SB [47] for OpB, using sander and PMEMD modules from Amber12 software (University of California, San Francisco, CA, USA).

To evaluate the results of molecular dynamics, we used the root-mean-square deviation (RMSD) of atomic positions that represent the conformational differences and fluctuations of all molecules during the molecular dynamics, using the CPPTRAJ module from Amber12 [48]. The free binding energy was also calculated using the MM-PBSA method, and the energy was decomposed by residues to see which residues contributed more to the stability of the complex [36,37].

### 4.4. Plant Material

Stem barks were collected in a land area in the village of Ananin (12/13/2012), a municipality of Peixe-Boi, Pará State, Brazil (01°04′54.2″ S,47°18′58.6″W). The voucher specimen was deposited at the Herbarium of the Museu Paraense Emílio Goeldi under the record MG-Etn-00245.

### 4.5. Biological Material

The parasite used was *Leishmania (L.) amazonensis,* isolated from a human case from Ulianópolis, Pará State (MHOM/BR/2009/M26361). Cell lines from acute monocytic leukemia (THP-1; ATCC No. TIB 202) were purchased from the cell bank of Rio de Janeiro (BCRJ).

### 4.6. Phytochemical Studies

The powder from the barks underwent maceration with ethanol (1:10), and the macerated material was concentrated in a rotary evaporator until residue precipitation. The extract was then submitted to acid–base partition, yielding the neutral fraction (NF) and the alkaloid fraction (AF). The AF was subjected to further fractionation in the chromatography column using Sephadex LH-20 as the stationary phase. Subfraction F6AF was subjected to fractionation in high-performance liquid chromatography (column SunFire™ Prep. C18 OBD™, 5 μm; 19 × 150 mm, Waters Corporation, Milford, MA, USA), resulting in the isolation of flavopereirine. For identification of this alkaloid, the Alliance HPLC-DAD system Waters (column SunFire^TM^ C18 5 μm; 4.6 × 150 mm), LC-MS (WATERS^®^ ACQUITY TQD SYSTEM/ESI using 50 mm × 2.1 mm 1.7μm Acquity UPLC BEH^®^ C18 analytical column, Waters Corporation, Milford, MA, USA), and NMR Varian Mercury 300 (300 MHz, CD_3_OD, Varian, Oxford, UK) were used.

The ethanolic extract was subjected to extraction under reflux [49], yielding 4 fractions: hexane, dichloromethane, ethyl acetate, and methanol. The extract and its fractions were subjected to characterization in thin-layer chromatography (Silicycle, Quebec City, QC, Canada) (TLC; revelators: UV and Dragendorff’s reagent) and high-performance liquid chromatography with diode-array detection (HPLC-DAD, Manufacturer, Milford, MA, USA).

Flavopereirine: data from UV: λ 234.3, 288.8, 346.1, 385.4 nm; data from MS: [M + H]+ m/z 315.37, 297.65, 160.21, 122.27; NMR 1H (300 MHz, CD_3_OD): 8.76 (d, 9.0), 8.30 (d, 8.1), 9.16 (s), 8.87 (d, 6.3), 8.66 (d, 6.9), 8.36 (d, 7.8), 7.48 (t, 7.8), 7.74 (t, 6.9), 7.82 (d, 8.4), 2.99 (q, 7.5), 1.45 (t, 7.2).

### 4.7. Antipromastigote Assay

Field isolates of Leishmania amazonensis (MHOM/BR/2009/M26361) were obtained from the Instituto Evandro Chagas, Brazil. Promastigotes of *L. amazonensis* were isolated on NNN (Novy-Nicolle-Mcneal) blood slopes. Afterwards, the strains were subcultured and adapted to RPMI 1640 medium supplemented with 10% heat inactivated fetal bovine serum (Gibco^®^, Grand Island, NY, USA) and cultivated at 26 °C [50].

The logarithmic phase of growth of promastigote forms was adjusted to 5 × 10^6^ parasites/100 µL. The susceptibility assay was performed on 96-well plates. The extract, fraction, and alkaloids were assayed in triplicates on a concentration gradient (200–3.125 μg/mL). The negative control was assessed with only parasites and the incubation medium; the positive control was assayed with amphotericin B (25–0.3906 μg/mL). After 24 h, 48 h, and 72 h of incubation at 26 °C under 5% CO_2_ atmosphere, 10 μL of the tetrazolium salt ([3-(4,5-dimethylthiazol-2-yl)-2,5-diphenyltetrazolium bromide], 5 mg/mL) was added to each well, and the parasites were quantified in the enzyme-linked immunosorbent assay plate reader [50]. The IC_50_ was determined by linear regression (Graph Pad Prism version 6.01, GraphPad Software, San Diego, CA, USA).

### 4.8. Cell Viability Assay

Cell viability was determined by the MTT assay ([3-(4,5-dimethylthiazol-2-yl)-2,5-diphenyltetrazolium bromide]) [51]. THP-1 modified cells (4 × 10^5^ cells/0.1 mL) were grown in RPMI-1640 medium (Sigma Aldrich^®^, St Louis, MO, USA), supplemented with 5% fetal bovine serum, and kept under 5% CO_2_ atmosphere at 37 °C. The cells were treated with extracts, fractions, subfractions, and alkaloids in different concentrations (ranging from 7.8125 to 500 μg/mL). After 24 h, 48 h, and 72 h of further incubation, MTT (5.0 mg/mL) was added. The plate was incubated at 37 °C in an atmosphere of 5% CO_2_ for 4 h. Dimethyl sulfoxide was added to each well to solubilize formazan crystals. Optical density was determined at 490 nm (Stat Fax 2100 microplate reader, Awareness Technology, Inc, Palm City, FL, USA). Cell viability was expressed as a percentage of the control absorbance in the untreated cells after subtracting the appropriate background. Cytotoxic concentration (CC_50_) was determined by linear regression [52].

## 5. Conclusions

In summary, our results suggest that *Geissospermum vellosii,* especially its indole alkaloid, flavopereirine, was very promising against *Leishmania amazonensis*. Moreover, its IC50 is lower than that obtained for amphotericin B, the last-resort drug for leishmaniosis treatment. That may be related to its interaction with OpB, presented in the docking results and maintained throughout the entirety of the molecular dynamics via π-stacking intermolecular interaction, presenting a possible novel inhibitory mechanism never shown before, and it may prevent substrates reaching the catalytic site.

## Figures and Tables

**Table 1:** Yields and thin layer chromatography of *Geissospermum vellosii.*

Samples	Yield (%)	TLC	
		UV Light	Dragendorff
**EE**	2.0	+	+
NF	42.8	+	+
AF	27.5	+	+
FrHex	1.8	+	+
FrDcm	5.8	+	+
FrAcOET	2.2	+	+
FrMeOH	85.2	+	+

TLC, thin-layer chromatography; UV, ultraviolet rays; EE, Ethanol Extract; NF, Neutral Fraction; AF, Alkaloid Fraction; FrHex, Hexane Fraction; FrDcm, Dichloromethane Fraction; FrAcOET, Ethyl Acetate Fraction; FrMeOH, Methanol Fraction.

**Table 2:** The anti-promastigote activity of *G. Vellosii.*

Samples	IC_50_ (µg/mL) + SD		
	24 h	48 h	72 h
EE	50.25 ± 0.36	29.57 ± 0.83	14.71 ± 0.70
FrHex	84.31 ± 0.50	>200	>200
FrDcm	5.56 ± 0.70	20.85 ± 0.25	10.06 ± 0.80
FrAcOET	12.32 ± 0.69	82.06 ± 0.26	88.61 ± 0.20
FrMeOH	1.71 ± 0.15	3.75 ± 0.52	5.95 ± 0.66
NF	34.59 ± 0.83	21.33 ± 0.19	18.25 ± 0.91
AF	6.22 ± 0.25	10.19 ± 0.18	1.07 ± 0.24
F6AF	1.56 ± 0.16 *	31.50 ± 0.76 *	1.24 ± 0.15 *
Flavopereirine	0.23 ± 0.10 * (0.93 µM)	2.34 ± 0.50 * 9.3 µM	0.15 ± 0.06 * 0.61 µM
Amphotericin	0.42 ± 0.09 (0.45 µM)	1.79 ± 0.06 (1.94 µM)	0.35 ± 0.01 (0.30 µM)

EE, Ethanol Extract; NF: Neutral Fraction; AF: Alkaloid Fraction; FrHex: Hexane Fraction; FrDcm: Dichloromethane Fraction; FrAcOET: Ethyl Acetate Fraction; FrMeOH: Methanol Fraction; * *p* < 0.05. **Legend**: The control of the untreated and solvent control presented viability corresponding to 100%.

**Table 3:** Cytotoxicity (CC_50_) and selective index (SI) of *G. Vellosii.*

Samples	24 h		48 h		72 h	
	CC_50_ *	SI	CC_50_ *	SI	CC_50_ *	SI
EE	178.0 ± 0.36	13.1	581.1 ± 0.60	19.7	829.4 ± 1.09	56.4
NF	115.9 ± 0.35	3.4	514.4 ± 0.61	23.4	744.3 ± 0.23	32.4
AF	121.4 ± 0.29	19.5	535.4 ± 0.43	52.5	774.3 ± 0.47	723.7
F6AF	443 ± 0.45	147.8	625.7 ± 0.34	19.9	629.4 ± 0.91	508.0
Flavopereirine	225.5 ± 0.9 (910 µM)	976.2	533.3 ± 0.15 (2156 µM)	228.2	734.0 ± 0.86 (2968 µM)	4993.2
Amphotericin	272.7 ± 0.09 (295.1 µM)	655.5	584.6 ± 0.46 (632.6 µM)	326.6	637.7 ± 0.72 (690 µM)	1811.7

* Human monocytic leukemia cell line (THP-1), concentration (µg/mL); EE, Ethanol Extract; NF, Neutral Fraction; AF, Alkaloid Fraction.

**Table 4:** Theoretical properties calculated from Molinspiration website of all tested molecules.

	Properties	Antipain	Flavopereirine
Molecules			
tLogP		−3.01	0.90
TPSA		282.51	19.89
Molecular Weight		604.71	247.32
nON		16	2
nOHNH		13	1
nViolations		3	0
nRotB		19	1
Volume		563.14	233.44

tLogP, Theoretical Octanol-Water Partition Coefficient; TPSA, Total Polar Surface Area; nON, number of hydrogen bond acceptors; nOHNH, number of hydrogen bond donors; nViolations, number of violations of Lipinski’s rule of five [24]; nRotB, number of rotatable bonds.
